# Change in Viral Load during Antiviral Therapy Is Not Useful for the Prediction of Hearing Dysfunction in Symptomatic Congenital Cytomegalovirus Infection

**DOI:** 10.3390/jcm10245864

**Published:** 2021-12-14

**Authors:** Takumi Kido, Yuki Kyono, Shutaro Suga, Ruka Nakasone, Shinya Abe, Mariko Ashina, Hisayuki Matsumoto, Kenji Tanimura, Kandai Nozu, Kazumichi Fujioka

**Affiliations:** 1 Department of Pediatrics, Kobe University Graduate School of Medicine, Kobe 650-0017, Japan; tkido@med.kobe-u.ac.jp (T.K.); ykyono@med.kobe-u.ac.jp (Y.K.); sugashu@med.kobe-u.ac.jp (S.S.); nakasone@med.kobe-u.ac.jp (R.N.); sky.my.kh@gmail.com (S.A.); marikoa@med.kobe-u.ac.jp (M.A.); nozu@med.kobe-u.ac.jp (K.N.); 2 Department of Clinical Laboratory, Kobe University Hospital, Kobe 650-0017, Japan; hisa-mt@live.jp; 3 Department of Obstetrics and Gynecology, Kobe University Graduate School of Medicine, Kobe 650-0017, Japan; tanimurakenji@gmail.com

**Keywords:** congenital cytomegalovirus infection, antiviral therapy, hearing dysfunction, viral load

## Abstract

For symptomatic congenital cytomegalovirus infections (CCMVI), the usefulness of changes in viral load during valganciclovir (VGCV) treatment for the prediction of hearing dysfunction (HD) is unclear. To determine the utility of viral load change in the whole blood or urine for the prediction of HD, we performed a retrospective study to compare viral load changes during VGCV treatment between CCMVI infants with (*n* = 12) or without (*n* = 8) HD at six months of corrected age, whose blood and urine viral loads were measured continuously for eight weeks from April 2009 to December 2019. There was no significant difference in the changes in both the blood and urine viral loads after the initiation of VGCV treatment between CCMVI infants between the groups. Moreover, this negative result was maintained in the analysis for each six weeks or six months treatment period. In conclusion, the change in viral load during antiviral therapy is not useful for the prediction of HD at six months of corrected age in symptomatic CCMVI.

## 1. Introduction

Congenital cytomegalovirus infection (CCMVI) is a disease that occurs when a mother experiences CMV infection during pregnancy, leading to vertical transmission to the fetus, and is the main etiology of congenital central nervous system disorders in developed countries. CMV is the most common cause of viral infections that spread mother-to-child, with a reported prevalence in 0.2–2.0% of pregnancies [1]. Since human CMV is a ubiquitous human-specific DNA virus, detection of CMV DNA in urine within three weeks after birth has become the gold standard for the diagnosis of CCMVI in recent years [1]. For long-term outcomes, approximately 70–90% of infants with symptomatic CCMVI, which is accompanied by any clinical symptoms at birth, develop neurological sequelae, including hearing dysfunction, chorioretinitis, neuromuscular disorders (epilepsy, hemiplegia, and convulsions), psychomotor impairment, and intellectual disability. Therefore, antiviral therapies such as intravenous ganciclovir (GCV) and oral valganciclovir (VGCV), which have been reported to improve the prognosis, have been widely investigated [2,3]. However, these antiviral treatments for newborns have been reported to have serious side effects, such as neutropenia, and do not have proven safety profiles [4]. Regarding treatment responsiveness of CCMVI, we have revealed that the most severe hearing dysfunction did not improve after VGCV treatment, although moderate or severe hearing dysfunction improved [5]. Thus, it is necessary to select the appropriate treatment target for antiviral therapy.

To elucidate antiviral treatment targets, many researchers, including ourselves, have conducted clinical studies on the prognostic factors for long-term outcomes in CCMVI. Concerning the symptoms at birth, we have clarified that the presence of microcephaly and/or being small for gestational age at birth, are risk factors for severe neurological sequelae with a developmental quotient (DQ) < 70 at 18 months of corrected age [6]. In terms of radiographic abnormalities, we have also reported that infants with CCMVI with at least two ventriculomegaly, periventricular cysts, and white matter abnormalities in early postnatal brain MRI images are at high risk of developing neurodevelopmental impairments at 18 months of corrected age [7]. Moreover, since the CMV-DNA titer reflects the viral burden in the body, there have been several reports examining the relationship between the viral load of CMV-DNA and neurodevelopmental outcomes [8,9,10].

In 2005, Boppana et al., reported that children with asymptomatic CCMVI infection with higher amounts of infectious CMV in urine and CMV-DNA viral load in the whole blood during early infancy are more likely to have hearing dysfunction, suggesting the role of viral burden and viral replication in the pathogenesis of CCMVI [8]. Similarly, Lanari et al., examined 44 CCMVI infants using qPCR and revealed that the mean values of viral load in polymorphonuclear leukocyte (PMNL) samples in the neonatal period were significantly higher in new-borns who developed sequelae at 12 months of age than in those who did not. They concluded that low blood CMV viral load in the neonatal period strongly predicts survival without sequelae, and the risk of developing sequelae was high when the blood CMV-DNA titers were ≥10,000 copies/10^5^ polymorphonuclear leukocytes [9]. Meanwhile, Forner et al. analyzed 33 infants with asymptomatic CCMVI and found that if the viral load of CMV DNA in the whole blood at birth was ≥12,000 copies/mL, the risk of developing postnatal sequelae exceeded 50%, and when the viral load of CMV DNA in the blood at birth was ≥17,000 copies/mL, the risk of hearing dysfunction exceeded 50% [10].

However, most of these studies used the results of a single viral load measurement, and the relationship between changes in viral load during antiviral treatment and neurological sequelae has not been fully elucidated. Therefore, since the usefulness of changes in viral load during VGCV treatment for the prediction of hearing dysfunction in symptomatic CCMVI is unclear, we performed a retrospective study to compare the viral load changes between CCMVI infants with or without hearing dysfunction at six months of corrected age.

## 2. Materials and Methods

### 2.1. Study Design and Patients

As part of a prospective cohort study for universal CMV screening at Kobe University Hospital [11,12,13], we measured whole blood, plasma, and urine viral loads by qRT-PCR in neonates suspected of having CCMVI. The study was conducted with approval from the Ethics Committee of Kobe University Graduate School of Medicine (#923), and written informed consent was obtained from all participants.

To determine the usefulness of changes in CMV viral load in the whole blood or urine for the prediction of hearing dysfunction in CCMVI post-VGCV treatment, we included CCMVI infants treated with oral VGCV treatment during a 10-year study period (April 2009 to December 2019), whose blood and urine viral loads were measured continuously for 8 weeks (from pre-treatment to 7 weeks post-treatment). The exclusion criteria were infants with other congenital abnormalities, infants with incomplete VGCV therapy, and infants with multiple viral load data deficiencies (>1).

CCMVI was definitively diagnosed by positive qRT-PCR results for urine obtained within 3 weeks after birth [12,14,15,16]. The urine and whole blood viral loads were measured by qRT-PCR, based on our previous report [16]. Clinical characteristics, including gestational age, birth weight, outborn status, sex, neonatal asphyxia, thrombocytopenia, liver dysfunction, microcephaly, hearing dysfunction, abnormalities on brain imaging (Supplementary Table S1), eye complications, small for gestational age (SGA), and symptomatic CCMVI, were retrospectively collected from electronic patient charts [17].

ABR was measured using the Neuropack S1 system (Nihon Kohden Co., Tokyo, Japan). According to our previous report, we tested the ability of the stimuli at amplitudes of 90 dB, 60 dB, and 30 dB to evoke ABR. Hearing status was classified as “normal” if the auditory stimulus evoked a detectable wave V at 30 dB. However, if wave V was undetectable at a given loudness, the test was repeated with a stimulus 10 dB higher in amplitude. The wave V threshold was defined as the lowest amplitude to evoke the ABR. The level of ABR abnormality was defined as a V-wave threshold and categorized as: most severe, ≥91 dB; severe, 61–90 dB; moderate, 41–60 dB; and mild, 31–40 dB. Hearing dysfunction (HD) was diagnosed at 6 months of corrected age, based on a V-wave threshold of >30 dB in at least one ear [5]. Thus, the patients with unilateral hearing dysfunctions were included in the HD group. In cases where test results were available, ABR results at 12 months or above were also examined.

Based on the presence or absence of HD, we classified the participants into two groups: the hearing dysfunction (HD) and the non-HD group. The changes in CMV viral load in the whole blood and urine were compared between the groups.

### 2.2. Measuring CMV Viral Load in the Blood and Urine

Using a previously reported method [16], DNA was extracted from the peripheral blood and urine using QIAamp DNA Mini kits (Qiagen Corp., Tokyo, Japan). CMV-DNA copy number was measured using real-time quantitative PCR. CMV DNA in whole blood was expressed in terms of copies/mL with a negative cut-off of 3 × 10^3^ copies/mL. CMV DNA in urine was expressed in terms of copies/mL with a negative cut-off of 3 × 10^3^ copies/mL [16].

### 2.3. VGCV Treatment Protocols

Symptomatic infants with congenital CMV infection were hospitalized for the first 6 weeks of VGCV therapy to monitor drug effects and adverse events. Depending on their condition, the patients were released to continue treatment in an outpatient setting after 6 weeks. The participants enrolled from November 2009 to June 2015 were administered oral VGCV for 6 weeks (32 mg/kg/d), whereas those enrolled from July 2015 onward were administered the same dose for 6 months [5,6,7]. Infants were regularly monitored for treatment effects and adverse events. A neonatologist examined the infants daily during the first 6 weeks of treatment, and their blood and urine CMV loads were measured once a week during the first 6 weeks of treatment and at least once monthly thereafter. Neutropenia (i.e., neutrophil count <500/mm^3^) is a common side effect of VGCV [2]; when observed, doctors temporarily discontinued treatment and waited for the neutrophil count to recover before resuming treatment. In this event, the duration of treatment (6 weeks or 6 months) was measured from the resumption of VGCV administration. If this took more than one week, G-CSF was administered, or treatment was resumed at a reduced dosage (16 mg/kg/d) for some patients, based on the attending physician’s discretion. We obtained written informed consent and approval from the ethics committee of the Kobe University Graduate School of Medicine (#1214).

### 2.4. Statistical Analysis

Data are expressed as median (range) or number (percentage). The Mann–Whitney nonparametric rank test, Fisher’s exact test, or two-way ANOVA test, as appropriate, were used to compare data between the two groups. Regression analysis was performed to linearly compare the whole blood and urine viral load, and regression equations and correlation coefficients (r^2^) were calculated. All analyses were performed using the GraphPad Prism 7 software (GraphPad Software, Inc., San Diego, CA, USA). Differences were considered statistically significant at *p* < 0.05.

## 3. Results

### 3.1. Patient Characteristics

Throughout the study period, 29 infants were diagnosed with symptomatic CCMVI and treated with oral VGCV treatment. Subsequently, two infants with other congenital abnormalities, two infants with VGCV therapy incompletion due to neutropenia, and five infants with multiple deficiencies of viral load data were excluded; thus, 20 infants were enrolled in this study. Among the enrolled infants, 12 had hearing dysfunction (HD) and 8 did not have hearing dysfunction (non-HD) at 6 months of corrected age.

Patient characteristics are shown in Table 1. There were no significant differences in clinical characteristics including blood and urine CMV viral load between the groups. Regarding the severity of auditory brainstem response (ABR) abnormalities, the normal was significantly lower in the ears of HD patients than in non-HD patients before VGCV treatment, at 6 months of corrected age, and at 12–18 months of corrected age (*p* < 0.05, respectively), whereas the most severe was significantly higher in the ears of HD patients than in non-HD patients both at 6 months of corrected age and at 12–18 months of corrected age (*p* < 0.05, respectively). In addition, the moderate was significantly higher in the ears of HD patients than in non-HD patients only at 12–18 months of corrected age.

### 3.2. Changes of Viral Load in the Whole Blood and Urine

**Figure 1:**
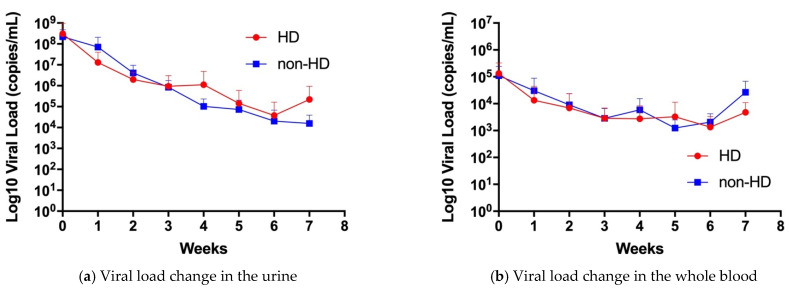
CMV viral load change in the urine and whole blood. (**a**) Viral load change in the urine, *n* = 12 in HD group (1 missing data in week 7), and *n* = 8 in non-HD group (2 missing data in week 7). (**b**) Viral load change in the whole blood, *n* = 12 in HD group (1 missing data in week 7), and *n* = 8 in non-HD group (1 missing data in week 1 and 3 missing data in week 7). Red circle symbols represent HD group and Blue square symbols represents non-HD groups. Data are shown as mean ± standard deviation. HD; hearing dysfunction, non-HD; non-hearing dysfunction.

We compared the change in viral load in the whole blood and urine between the HD and non-HD groups using two-way ANOVA (Figure 1). There was no significant difference in viral load change between the HD and non-HD groups in the urine (*p* = 0.91, Figure 1a) and whole blood (*p* = 0.82, Figure 1b), respectively.

### 3.3. Changes of Viral Load in 6 Weeks and 6 Months VGCV Treatment

**Figure 2:**
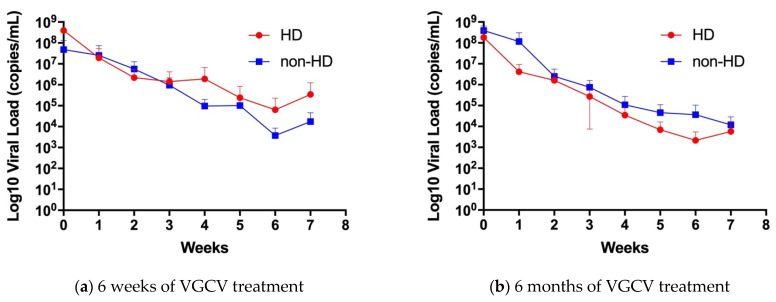
CMV viral load change in urine of (**a**) 6 weeks or (**b**) 6 months of VGCV treatment groups. (**a**) Urine viral load change over 6 weeks of VGCV treatment, *n* = 7 in HD group, and *n* = 4 in non-HD group. (**b**) Urine viral load change over 6 months of VGCV treatment, *n* = 5 in HD group (1 missing data in week 7), and *n* = 4 in non-HD group (2 missing data in week 7). Red circle symbols represent HD group and Blue square symbols represents non-HD groups. Data are shown as mean ± standard deviation. HD; hearing dysfunction, non-HD; non-hearing dysfunction.

We then divided the infants into two groups based on the duration of VGCV treatment: 6 weeks and 6 months of treatment. First, we compared the change in viral load in urine between the HD and non-HD groups at 6 weeks and 6 months, respectively (Figure 2). There was no significant difference in the urine viral load change between the HD and non-HD groups in 6 weeks treatment group (*p* = 0.48, Figure 2a) and 6 months treatment group (*p* = 0.08, Figure 2b), respectively.

**Figure 3:**
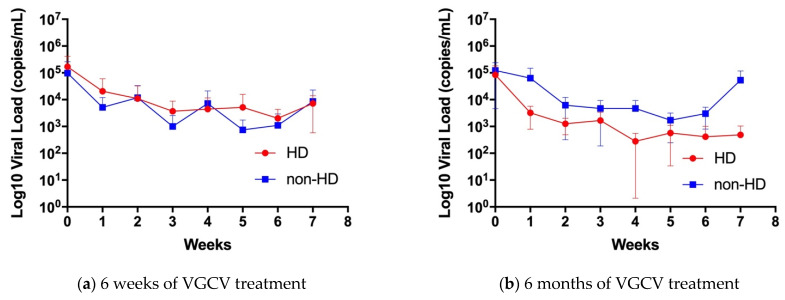
CMV viral load change in the whole blood of 6 weeks or 6 months of VGCV treatment groups. (**a**) Blood viral load change over 6 weeks of VGCV treatment, *n* = 7 in HD group, and *n* = 4 in non-HD group (1 missing data in week 7). (**b**) Blood viral load change over 6 months of VGCV treatment, *n* = 5 in HD group (1 missing data in week 7), and *n* = 4 in non-HD group (1 missing data in week 1 and 2 missing data in week 7). Red circle symbols represent HD group and Blue square symbols represents non-HD groups. Data are shown as mean ± standard deviation. HD; hearing dysfunction, non-HD; non-hearing dysfunction.

Second, we compared the change in viral load in the blood between the HD and non-HD groups at 6 weeks and 6 months (Figure 2). There was no significant difference in the blood viral load between the HD and non-HD groups in the 6 weeks treatment group (*p* = 0.53, Figure 3a) and 6 months treatment group (*p* = 0.06, Figure 3b), respectively.

### 3.4. Correlation between the Urine and Whole Blood Viral Load

**Figure 4:**
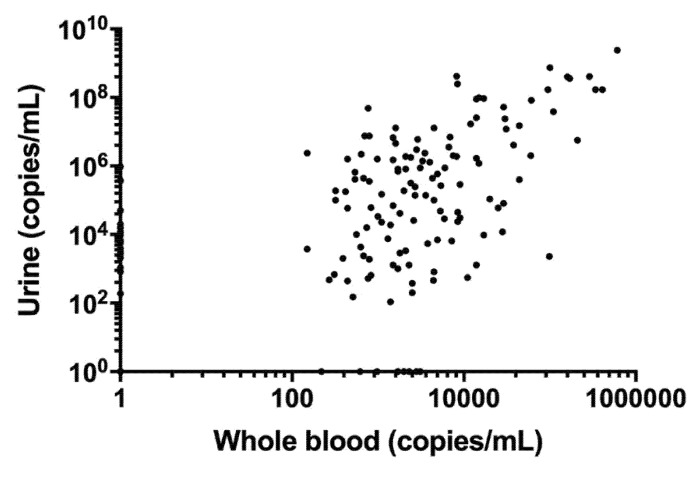
Correlation of viral loads in the whole blood and urine. Blood viral load significantly correlated with the urine viral load (*n* = 155; y = 2198x − 8561549, r^2^ = 0.579, *p* < 0.0001).

Subsequently, we calculated the correlation between the whole blood and urine viral loads. The correlation of viral loads in the whole blood and urine is shown in Figure 4. Whole blood viral loads were significantly correlated with urine viral loads (y = 2198x − 8561549, r^2^ = 0.579, *p* < 0.0001).

## 4. Discussion

In this study, we found no significant difference in the changes in the blood and urine viral loads after the initiation of VGCV treatment between CCMVI infants with and without HD at 6 months of corrected age. Additionally, this negative result was maintained in the analyses during each six-week or 6-month treatment period.

To date, there have been only two conflicting studies regarding the changes in viral load and long-term outcomes in CCMVI. The first was a prospective observational study with nine CCMVI patients who received oral VGCV treatment for six weeks, as reported by Kawata et al. They found that CMV-DNA in the blood decreased to less than the detection limit one week after the initiation of antiviral therapy in four patients who did not develop HD at one year of age, whereas CMV-DNA was detected in the blood continuously or intermittently in five patients with HD. They concluded that the long-term detection of blood CMV during antiviral therapy could be a risk factor for SNHL [18]. However, the number of patients enrolled in their study was less than half that in our study. Thus, they could only perform repeated single-point comparisons (Mann–Whitney nonparametric rank test), but not continuous variable comparisons (two-way ANOVA test) as we performed. We believe that continuous variable comparison, as we adopted, is necessary to examine continuous changes in viral load.

The second study was a post hoc analysis of two clinical trials of 6 months of VGCV treatment for CCMVI performed by Marsico et al. In that study, the authors found that sustained viral suppression during six months of therapy correlated with better hearing outcomes at six, 12, and 24 months of age; however, a majority of their patients without viral suppression still exhibited improved hearing. Thus, they concluded that the viral load at baseline or during treatment should not be considered as a marker to assess the effectiveness of antiviral therapy in CCMVI [19]. Although their study included more patients than ours, the changes in viral load were not examined over time on a weekly basis. Despite the different study designs, theirs and our conclusions agree regarding the changes in the CMV viral load during VGCV treatment not being clinically significant.

In addition, we found that several patients included in the non-HD group showed improvement in hearing level after VGCV treatment. This result is consistent with our previous report that 55% of all treated ears and 84% of ears with moderate or severe hearing dysfunction improved after treatment [5].

A limitation of the present study is that the number of patients was relatively small due to its retrospective nature. Moreover, HD was evaluated at six months of corrected age, and the relationship with long-term hearing prognosis such as that at one or two years was not examined in this study. However, in a retrospective study that analyzed the stability of ABR abnormality during the first year of life, a relatively low percentage of instability of results in the groups of children with CCMVI was reported [20]. In addition, the results of our subjects with ABR data at 12 months or above available were in good agreement with the results at six months. Thus, we believe our HD groups would have poor long-term hearing outcomes. In the future, prospective studies comparing the long-term auditory prognosis and viral load change are needed.

## 5. Conclusions

The change in viral load during antiviral therapy is not useful for the prediction of HD at six months of corrected age in symptomatic CCMVI patients.

## Figures and Tables

**Table 1:** Clinical characteristics of infants with and without CCMVI diagnoses confirmed by PCR.

Clinical Characteristics	HD Group (*n* = 12)	non-HD Group (*n* = 8)	*p* Value
Gestational age, weeks	37 (33–40)	36 (30–40)	0.61
Birth weight, g	2261 (1255–3312)	2321 (940–2848)	0.91
Outborns	5/12 (42)	3/8 (38)	0.67
Male	3/12 (25)	2/8 (25)	1.00
Neonatal asphyxia $	5/10 (50)	4/8 (50)	1.00
Thrombocytopenia	5/12 (42)	5/8 (63)	0.65
Liver dysfunction	4/12 (33)	2/8 (25)	1.00
Microcephaly	4/12 (33)	2/8 (25)	1.00
Brain imaging abnormality	12/12 (100)	8/8 (100)	1.00
Eye complications	2/12 (17)	3/8 (38)	0.35
Small for gestational age	5/12 (42)	2/8 (25)	0.64
CMV load in blood before VGCV treatment, copies/mL	3.7 × 10^4^ (7.7 × 10^2^–6.1 × 10^5^)	6.7 × 10^4^ (1.5 × 10^3^–3.4 × 10^5^)	0.97
CMV load in urine before VGCV treatment, copies/mL	8.9 × 10^7^ (4.0 × 10^5^–2.4 × 10^9^)	9.8 × 10^8^ (1.5 × 10^6^–4.1 × 10^8^)	0.52
ABR abnormality before VGCV treatment	(*n* = 24 ears)	(*n* = 16 ears)	
most severe (≥91 dB)	7/24 (29)	1/16 (6)	0.11
severe (61–90 dB)	5/24 (21)	1/16 (6)	0.37
moderate (41–60 dB)	3/24 (13)	4/16 (25)	0.40
mild (31–40 dB)	5/24 (21)	1/16 (6)	0.37
normal (≤30 dB)	4/24 (17)	9/16 (56)	0.02
ABR abnormality at 6 months of corrected age	(*n* = 24 ears)	(*n* = 16 ears)	
most severe (≥91 dB)	7/24 (29)	0/16 (0)	0.03
severe (61–90 dB)	3/24 (13)	0/16 (0)	0.26
moderate (41–60 dB)	6/24 (25)	0/16 (0)	0.06
mild (31–40 dB)	5/24 (21)	0/16 (0)	0.07
normal (≤30 dB)	3/24 (13)	16/16 (100)	<0.01
ABR abnormality at 12–18 months of corrected age #	(*n* = 16 ears)	(*n* = 14 ears)	
most severe (≥91 dB)	5/16 (31)	0/14 (0)	0.04
severe (61–90 dB)	1/16 (6)	0/14 (0)	1.00
moderate (41–60 dB)	6/16 (38)	0/14 (0)	0.02
mild (31–40 dB)	0/16 (0)	1/14 (7)	0.47
normal (≤30 dB)	4/16 (25)	13/14 (93)	<0.01

Data are shown as median (range) or number (percentage). HD, hearing dysfunction; non-HD, non-hearing dysfunction; ABR, auditory brainstem response. $ Data were not available for 2 infants in HD group. # Data were not available for 4 infants in HD group and 1 infant in non-HD group.

## Data Availability

The data presented in this study are available in this article.

## Supplementary Materials

The following are available online at https://www.mdpi.com/article/10.3390/jcm10245864/s1, Table S1: Detailed findings of brain imaging (ultrasonography, computed tomography, and magnetic resonance imaging).

## References

[1] Rawlinson W.D., Boppana S.B., Fowler K.B., Kimberlin D.W., Lazzarotto T., Alain S., Daly K., Doutre S., Gibson L., Giles M.L. (2017). Congenital cytomegalovirus infection in pregnancy and the neonate: Consensus recommendations for prevention, diagnosis, and therapy. Lancet Infect. Dis..

[2] Kimberlin D.W., Lin C.Y., Sanchez P.J., Demmler G.J., Dankner W., Shelton M., Jacobs R.F., Vaudry W., Pass R.F., Kiell J.M. (2003). Effect of ganciclovir therapy on hearing in symptomatic congenital cytomegalovirus disease involving the central nervous system: A randomized, controlled trial. J. Pediatr..

[3] Kimberlin D.W., Jester P.M., Sanchez P.J., Ahmed A., Arav-Boger R., Michaels M.G., Ashouri N., Englund J.A., Estrada B., Jacobs R.F. (2015). Valganciclovir for symptomatic congenital cytomegalovirus disease. N. Engl. J. Med..

[4] Ziv L., Yacobovich J., Pardo J., Yarden-Bilavsky H., Amir J., Osovsky M., Bilavsky E. (2019). Hematologic Adverse Events Associated With Prolonged Valganciclovir Treatment in Congenital Cytomegalovirus Infection. Pediatr. Infect. Dis. J..

[5] Ohyama S., Morioka I., Fukushima S., Yamana K., Nishida K., Iwatani S., Fujioka K., Matsumoto H., Imanishi T., Nakamachi Y. (2019). Efficacy of Valganciclovir Treatment Depends on the Severity of Hearing Dysfunction in Symptomatic Infants with Congenital Cytomegalovirus Infection. Int. J. Mol. Sci..

[6] Fukushima S., Morioka I., Ohyama S., Nishida K., Iwatani S., Fujioka K., Mandai T., Matsumoto H., Nakamachi Y., Deguchi M. (2019). Prediction of poor neurological development in patients with symptomatic congenital cytomegalovirus diseases after oral valganciclovir treatment. Brain Dev..

[7] Nishida K., Fujioka K., Sugioka Y., Abe S., Ashina M., Fukushima S., Ohyama S., Ikuta T., Tanimura K., Yamada H. (2020). Prediction of Neurodevelopmental Impairment in Congenital Cytomegalovirus Infection by Early Postnatal Magnetic Resonance Imaging. Neonatology.

[8] Boppana S.B., Fowler K.B., Pass R.F., Rivera L.B., Bradford R.D., Lakeman F.D., Britt W.J. (2005). Congenital cytomegalovirus infection: Association between virus burden in infancy and hearing loss. J. Pediatr..

[9] Lanari M., Lazzarotto T., Venturi V., Papa I., Gabrielli L., Guerra B., Landini M.P., Faldella G. (2006). Neonatal cytomegalovirus blood load and risk of sequelae in symptomatic and asymptomatic congenitally infected newborns. Pediatrics.

[10] Forner G., Abate D., Mengoli C., Palu G., Gussetti N. (2015). High Cytomegalovirus (CMV) DNAemia Predicts CMV Sequelae in Asymptomatic Congenitally Infected Newborns Born to Women With Primary Infection During Pregnancy. J. Infect. Dis..

[11] Tanimura K., Tairaku S., Morioka I., Ozaki K., Nagamata S., Morizane M., Deguchi M., Ebina Y., Minematsu T., Yamada H. (2017). Universal Screening With Use of Immunoglobulin G Avidity for Congenital Cytomegalovirus Infection. Clin. Infect. Dis..

[12] Tanimura K., Yamada H. (2018). Potential Biomarkers for Predicting Congenital Cytomegalovirus Infection. Int. J. Mol. Sci..

[13] Tanimura K., Yamada H. (2019). Maternal and neonatal screening methods for congenital cytomegalovirus infection. J. Obstet. Gynaecol. Res..

[14] Nishida K., Morioka I., Nakamachi Y., Kobayashi Y., Imanishi T., Kawano S., Iwatani S., Koda T., Deguchi M., Tanimura K. (2016). Neurological outcomes in symptomatic congenital cytomegalovirus-infected infants after introduction of newborn urine screening and antiviral treatment. Brain Dev..

[15] Koyano S., Inoue N., Oka A., Moriuchi H., Asano K., Ito Y., Yamada H., Yoshikawa T., Suzutani T. (2011). Japanese Congenital Cytomegalovirus Study Group. Screening for congenital cytomegalovirus infection using newborn urine samples collected on filter paper: Feasibility and outcomes from a multicentre study. BMJ Open.

[16] Kobayashi Y., Morioka I., Koda T., Nakamachi Y., Okazaki Y., Noguchi Y., Ogi M., Chikahira M., Tanimura K., Ebina Y. (2015). Low total IgM values and high cytomegalovirus loads in the blood of newborns with symptomatic congenital cytomegalovirus infection. J. Perinat. Med..

[17] Ohyama S., Fujioka K., Fukushima S., Abe S., Ashina M., Ikuta T., Nishida K., Matsumoto H., Nakamachi Y., Tanimura K. (2019). Diagnostic Value of Cytomegalovirus IgM Antibodies at Birth in PCR-Confirmed Congenital Cytomegalovirus Infection. Int. J. Mol. Sci..

[18] Kawada J., Torii Y., Kawano Y., Suzuki M., Kamiya Y., Kotani T., Kikkawa F., Kimura H., Ito Y. (2015). Viral load in children with congenital cytomegalovirus infection identified on newborn hearing screening. J. Clin. Virol..

[19] Marsico C., Aban I., Kuo H., James S.H., Sanchez P.J., Ahmed A., Arav-Boger R., Michaels M.G., Ashouri N., Englund J.A. (2019). Blood Viral Load in Symptomatic Congenital Cytomegalovirus Infection. J. Infect. Dis..

[20] Kocon S., Skorkiewicz K., Strek P., Ziarno R., Skladzien J., Hartwich P., Tomik J. (2020). Stability of ABR Wave V Threshold in Early Hearing Diagnostics in Children from Selected Groups at Risk of Congenital Hearing Loss. Otolaryngol. Pol..

