# Supplementary material for: Change in Viral Load during Antiviral Therapy Is Not Useful for the Prediction of Hearing Dysfunction in Symptomatic Congenital Cytomegalovirus Infection
Source: J Clin Med. 2021 Dec 14;10(24):5864. doi: 10.3390/jcm10245864 (PMC8709202; doi:10.3390/jcm10245864)
Supplement: Supplementary file 1 [file jcm-10-05864-s001.zip › jcm-1499035-supplementary.pdf]

Supplementary Table S1. Detailed findings of brain imaging (ultrasonography, computed tomography, and magnetic resonance imaging)

Clinical Characteristics	HD group (n = 12)	non-HD group (n = 8)	P value
Ventriculomegaly	12/12 (100)	5/8 (63)	0.049
Periventricular cysts	7/12 (58)	5/8 (63)	1.00
Cerebellar hypoplasia	1/12 (8)	0/8 (0)	1.00
Migration disorders	5/12 (42)	1/8 (13)	0.32
White matter abnormalities	3/12 (25)	4/8 (50)	0.36
Calcification	6/12 (50)	5/8 (63)	0.67

Data are shown as number (percentage). Fisher's exact test were used to compare data between the two groups. HD, hearing dysfunction; non-HD, non-hearing dysfunction.
